# A European Multicentric Investigation of Atypical Melanocytic Skin Lesions of Palms and Soles: The *iDScore-PalmoPlantar* Database

**DOI:** 10.3390/diagnostics14050460

**Published:** 2024-02-20

**Authors:** Linda Tognetti, Alessandra Cartocci, Aimilios Lallas, Elvira Moscarella, Ignazio Stanganelli, Gianluca Nazzaro, John Paoli, Maria Concetta Fargnoli, Paolo Broganelli, Harald Kittler, Jean-Luc Perrot, Gennaro Cataldo, Gabriele Cevenini, Sofia Lo Conte, Leonardelli Simone, Elisa Cinotti, Pietro Rubegni

**Affiliations:** 1 Dermatology Unit, Department of Medical, Surgical and Neurosciences, University of Siena, 53100 Siena, Italy; 2 First Department of Dermatology, Aristotle University, 54124 Thessaloniki, Greece; 3 Dermatology Unit, University of Campania Luigi Vanvitelli, 81100 Naples, Italy; 4 Skin Cancer Unit, Scientific Institute of Romagna for the Study of Cancer, Istituti di Ricovero e Cura a Carattere Scientifico (IRCCS), Istituto Tumori della Romagna (IRST), 47014 Meldola, Italy; 5 Department of Dermatology, University of Parma, 43121 Parma, Italy; 6 Fondazione IRCCS Ca’ Granda Ospedale Maggiore Policlinico, 20122 Milan, Italy; 7 Department of Dermatology and Venereology, Institute of Clinical Sciences, Sahlgrenska Academy, University of Gothenburg, 41390 Gothenburg, Sweden; 8 Department of Dermatology and Venereology, Region Västra Götaland, Sahlgrenska University Hospital, 41345 Gothenburg, Sweden; 9 Dermatology Unit, University of L’Aquila, 67100 L’Aquila, Italy; 10 Dermatology Unit, University Hospital of Torino, 4020 Torino, Italy; 11 Department of Dermatology, Medical University of Vienna, 1090 Vienna, Austria; 12 Dermatology Unit, University Hospital of St-Etienne, 42270 Saint Etienne, France; 13 Department of Medical Biotechnologies, University of Siena, 53100 Siena, Italy

**Keywords:** acral melanoma, acral nevi, dermoscopy, integrated dataset, web registry, atypical pigmented palmoplantar lesions

## Abstract

**Background:** The differential diagnosis of atypical melanocytic palmoplantar skin lesions (aMPLs) represents a diagnostic challenge, including atypical nevi (AN) and early melanomas (MMs) that display overlapping clinical and dermoscopic features. We aimed to set up a multicentric dataset of aMPL dermoscopic cases paired with multiple anamnestic risk factors and demographic and morphologic data. **Methods:** Each aMPL case was paired with a dermoscopic and clinical picture and a series of lesion-related data (maximum diameter value; location on the palm/sole in 17 areas; histologic diagnosis; and patient-related data (age, sex, family history of melanoma/sunburns, phototype, pheomelanin, eye/hair color, multiple/dysplastic body nevi, and traumatism on palms/soles). **Results:** A total of 542 aMPL cases—113 MM and 429 AN—were collected from 195 males and 347 females. No sex prevalence was found for melanomas, while women were found to have relatively more nevi. Melanomas were prevalent on the heel, plantar arch, and fingers in patients aged 65.3 on average, with an average diameter of 17 mm. Atypical nevi were prevalent on the plantar arch and palmar area of patients aged 41.33 on average, with an average diameter of 7 mm. **Conclusions:** Keeping in mind the risk profile of an aMPL patient can help obtain a timely differentiation between malignant/benign cases, thus avoiding delayed and inappropriate excision, respectively, with the latter often causing discomfort/dysfunctional scarring, especially at acral sites.

## 1. Introduction

Acral-pigmented lesions are still less investigated by dermoscopy than facial or body-pigmented lesions, and the referring terminology is otherwise rather confused [1,2,3,4,5]. To date, the term “acral” has been used to define melanocytic lesions localized not only on volar glabrous skin surfaces of the extremities but also on the nail apparatus and subungual region—especially in reference to acral lentiginous melanoma (MM) [1,2,3,4,5]. Moreover, in many different studies to date, the terms “acquired acral nevi”, “congenital acral nevi”, “acral melanocytic lesions”, and “acral lentiginous melanoma” have been vaguely employed, without specifying if the lesions were on palms/soles or subungual region/nail. However, the acral glabrous skin, which is anatomically limited to the palms of the hands and soles of the feet, distally to the Wallace’s line, significantly differs from other body sites skin areas, both clinically and dermoscopically, due to the presence of dermatoglyphics [1,2,3,4,5,6,7]. To avoid confusion, in this study we preferred naming the melanocytic lesion on glabrous acral skin as “melanocytic (M) palmoplantar (PP) lesions (Ls)” (MPPLs). This definition encompasses the spectrum of histologically benign MPPLs (with no/mild/moderate/severe atypia), histologically malignant MPPLs, and the grey zone of borderline provisional entities such as nevi MELTUMP/SAMPU/THIUMP/IAMPUS lesions [6].

The prevalence of MPPLs varies greatly according to populations, countries, and study groups, and it is essentially in line with that of benign MPPLs, ranging from 36–42% in dark phototypes and 18–23% in Caucasians [4,5,6,7]. This may also explain why studies focused on large datasets of acral nevi in Europe are scarce [8,9]. Interestingly, in dark skin types and Asiatic populations, the number of PP nevi is relatively high compared with other body sites and in a globally low total-body nevus count, while the trend is opposite in European and North American populations [1,2,3,4,5,6,7]. In parallel, the current bulk of knowledge from PP melanoma greatly derives from studies carried out in Asiatic countries (e.g., Japan, China, Taiwan) in the last 30 years [10,11,12,13], where this form accounts for nearly 50% of all MM cases. On the contrary, PP melanoma is traditionally considered rare in Caucasians, accounting for 3% of all MMs in North America and about 1–2% in Europe [1,3,9,11]. There is currently no univocal hypothesis to explain these discrepancies of both benign and malignant MPPLs among different populations: genetic predisposition is known to play a role, but genetic studies recently highlighted that PP nevi exhibit a mutational spectrum comparable to that of nevi arising on low cumulative sun-damaged skin [1,14,15]. Currently, PP melanoma is regarded as a non-UV-related tumor and represents a higher proportion of cases in countries with a lower incidence of melanoma overall [1]. External mechanisms and risk factors such as trauma, physical stress, and friction have been hypothesized to have a role in its development, but no conclusive data have been produced to date [16,17,18]. Additionally, other factors have been addressed such as the rarely examined location (sole melanoma), the atypical appearance (palm melanoma), and the lack of pigment (PP melanoma) [19,20].

Dermoscopic examination was shown to help increase the diagnostic accuracy of MPPLs, especially in differentiating malignant from benign cases [21,22] and in clear-cut lesions. Indeed, considering clear-cut PP nevi and PP melanomas, a series of specific dermoscopic patterns were first described by Japanese study groups and included benign-related features (parallel furrow pattern, lattice-like, fibrillar, globular, and homogenous) and malignancy-related features (parallel-ridge pattern, irregular diffuse pigmentation, and multicomponent pattern) [7,9,11,23,24,25]. We have otherwise to keep in mind that there are atypical MPPLs (aMPPLs) that exhibit equivocal clinical and dermoscopic features, including PP nevi mimicking PP melanomas (e.g., asymmetrical, maculopapular, and with non-homogenous pigmentation) and, vice versa, featureless or doubtful early melanoma [1,10,13,26]. In this subset of difficult “borderline” lesions, dermoscopy alone cannot reach adequate diagnostic accuracy, and further parameters should be taken into account to assess the risk of that lesion being malignant [24,25,26]. It has been widely demonstrated that the Bayesian scoring classifier models are reliable tools able to efficiently select and combine a series of patient and lesion objective parameters with dermoscopic data, with the final aim of developing a risk scoring model dedicated to a specific subset of lesions [27,28,29,30,31,32,33]. In particular, our group previously created and tested four different risk scoring models, named “integrated clinic-dermoscopic scores” (*iDScore*) for difficult-to-diagnose melanocytic skin lesions of the body (i.e., early melanomas and atypical nevi (AN) [28,29], for regressing nevi and melanomas with regression [30], and for atypical pigmented lesions of the face (i.e., lentigo maligna and benign simulators—pigmented actinic keratosis, solar lentigo, seborrheic keratosis, lichen planus-like keratosis, and atypical nevi) [31]. The development of an *iDScore* model relies, at first, on the preparation of a large detailed and standardized dataset of the lesions of interest. A dataset of 1700 cases of atypical melanocytic lesions of the body [32] and about 2000 cases of atypical pigmented lesions of the face [29] was developed, with each case integrated with multiple data of the patient and lesion and further subjected to pattern analysis and complex statistical analysis [28,29,30,31,32,33].

On these premises, we aimed to create, for the first time, a large international web registry able to provide a detailed characterization of aMPPLs (including early PP melanomas and atypical and/or dysplastic PP nevi) in terms of morphology (clinical and dermoscopic), epidemiology, patient risk factors, and anamnestic data. 

In this study, we describe the development and implementation of a European multicenter database specifically dedicated to aMPPLs, the *iDScore-PalmoPlantar* dataset.

## 2. Materials and Methods

***Ethics.*** This study was carried out in compliance with the Helsinki Declaration. Approval was obtained by the local ethical committee of Siena Hospital (Azienda Ospedaliero-Universitaria Senese, Siena, Italy, Study Protocol No. 16801) and was then shared with the participating centers. All data were de-identified before use and are kept in accordance with the EU General Data Protection Regulations (GDPR) on the processing of personal data and the protection of privacy in electronic communication (2016/679/EU) [34]. 

***Study design.*** The development of the international clinical–dermoscopic database dedicated to aMPPLs was promoted as part of the *iDScore-PalmoPlantar* project by dermatologists (LT, PR, and EC) and technical figures (bioengineer: GC, biostatisticians: AC and SLC, and data manager: GC) of Siena University Hospital and extended to the Teledermoscopy Working Group (AL, MCF, IS, GN, PB, JP, HK, JLP, EM, FL, CL, ED, MS, and EC) under the Teledermatology Task Force of the European Academy of Dermatology and Venereology (EADV). The *iDScore-PalmoPlantar* project is devoted to the study of difficult-to-diagnose melanocytic skin lesions from a clinical and dermoscopic point of view. In particular, the *iDScore-PalmoPlantar* database was designed for educational and training purposes, through a tele-dermoscopic setting, accessible to all European dermatologists; thus, the database is currently hosted on a dedicated website, www.iDScore.net (accessed on 16 February 2023).

***Center participation.*** A center was enrolled in the *iDScore-PalmoPlantar* project if it could provide at least 60 cases (up to a maximum of 110) of clinically and dermoscopically challenging aMPPLs excised in the suspect of malignancy. Thus, each center was required to provide a minimum of 20 malignant cases (up to 30) and a minimum of 40 benign cases (up to 80). Participation in the study was open to any European dermatology center actively working in skin cancer screening as a second-level referring center. The data were collected both retrospectively and prospectively: the collection phase lasted from September 2020 to March 2023. Since data were collected during routine consultation activity, there were neither costs nor financial compensation to participate. Each center designated one Site Investigator as responsible for the whole selection and submission process. Site Investigators were required to sign in to a web platform—hosted at www.iDScore.net—through secure access with personal credentials. Site Investigators were enabled to upload their cases from between October 2020 to June 2023 by using a “Contribution form” specifically created for the project and hosted on the website: the form was designed to record a total of 14 parameters (5 mandatory and 9 optional) along with 2 standardized image files. The assessment of the specific palmar or plantar location was mandatory; thus, site investigators were guided to select only one site per image among 9 areas of the palms or only one among 8 areas of the soles. 

**Figure 1:**
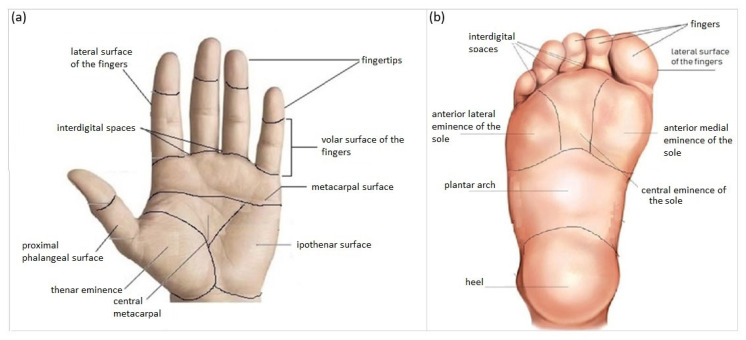
Schematic representation of the classification used in the *iDScore-PalmoPlantar* database into 17 areas: 9 palmar (**a**) and 8 plantar (**b**).

***Inclusion criteria.*** In order to avoid repetition of clinical/anamnestic data and thus bias affecting the analysis, each lesion had to be derived from one patient only. Each aMPPL case must be composed of one dermoscopic image, one clinical image, three mandatory lesion data (i.e., definitive histopathological diagnosis, maximum diameter (mm), and precise body location), and two mandatory patient data (i.e., sex (F/M) and age (years). Histologic diagnosis could be (a) nevus with mild atypia, (b) nevus with severe atypia, (c) dysplastic nevus, or (d) melanoma in situ or stage Ia/Ib/IIa (pathologic TNM classification pTis/pT1a/pT1b/pT2a). Additional histological data were required for MM cases only: thickness, mitosis number, regression (%), and presence of lymphocytic infiltrate. Patients were required to be aged at least 18 years; there was no upper range limit. According to anatomical and morphologic criteria, a classification into 17 subareas was adopted (Figure 1), including 8 plantar areas (i.e., *anterior lateral eminence of the sole, anterior medial eminence of the sole, central eminence of the sole, heel, interdigital spaces, lateral surface of the fingers, and plantar region)* and 9 volar palmar areas (i.e., *plantar surface of the fingers* of the sole, *central metacarpal, fingertips, interdigital spaces, hypothenar surface, lateral surface of the fingers, metacarpal surface, thenar surface* and *volar surface of the fingers, and proximal phalangeal surface)*.

***Patients’ additional data.*** Details concerning 4 anamnestic data and 5 phenotypic traits were strongly recommended (Table 1). Four types of anamnestic data were strongly recommended, though not mandatory, namely personal or family history of melanoma (i.e., in a 1st-degree relative), history of sunburns (>3) in childhood below the age of 14 years, history of chronic traumatism on the soles for work, and chronic traumatism on the palms for work. Five types of patient clinical data were strongly recommended, though not mandatory, namely presence of multiple common nevi (>100) or dysplastic nevi (>10) on the body, phototype (I–IV), pheomelanin phototype [35,36,37], presence of green/light blue/blue eyes, and presence of blond hair. In order to avoid repetition of clinical/anamnestic data and thus bias affecting the analysis, each lesion should be derived from one patient only. 

***Technical requirements.*** Each site investigator should also respect a series of technical requirements for the dermoscopic images (i.e., ≥1.5 Mpx, 15–20× enlargement, JPEG format, in-focus picture) and device type (e.g., videodermatoscope—Fotofinder system Medcam1000, camera-based systems—Dermlite Photo System Pro/Dermlite Foto II Pro WITH Nikon D500, 3GEN Dermlite Foto Dermoscopy System, Heine DL 20 Canon/Nikon, smartphone-based system—Foto X Dermlite).

***Exclusion criteria and quality check.*** Exclusion criteria for center contribution relied on the impossibility of reaching the adequate number and proportion of MMs and AN required. Exclusion criteria for pictures included blurred/out-of-focus dermoscopic pictures; clinical pictures with recognizable patient personal characteristics (e.g., tattoos, etc.); and nodular/ulcerated/inflamed/intensely traumatized MPPLs. Duplicate cases (e.g., multiple dermoscopic images of the same patient uploaded as separate cases or the same case entered 2 or 3 times) were rejected as well. Once uploaded onto the platform, each submission was examined; if judged suitable, the case was transferred to the *iDScore-PalmoPlantar* dataset itself. A review of all the cases received in the registry was performed weekly by LT, AC, GC, and SL from October 2020 to July 2023. This rapid review after each submission allowed all Site Investigators to be updated on their acceptance rate and allowed them to proceed with contributions until the minimum criteria were reached. 

***Statistical analysis.*** Descriptive statistics was carried out; continuous variables were summarized as mean ± standard deviation, with the qualitative ones recorded as absolute frequencies and percentages. The χ-squared test was performed to examine the association between qualitative variables and histological diagnosis. Student’s *t* test was performed to compare age and maximum diameter between MMs and AN. A significance of *p* < 0.05 was assumed. All analyses were carried out using R version 4.0.0. For statistical purposes, the 17 subareas were further grouped into 7 macro-areas, namely 3 macro-areas on the palm and 4 macro-areas on the sole (Table 1). 

## 3. Results

### 3.1. Participating Centers 

A total of 21 dermatologic centers from 14 European Countries were invited; all of them had a second-level ambulatory clinic active in screening and research on skin cancer. Among them, 10 were able to meet the minimum contribution criteria, namely Siena (Italy), Thessaloniki (Greece), Meldola (Italy), Milan (Italy), Gothenburg (Sweden), L’Aquila (Italy), Turin (Italy), Vienna (Austria), St. Etienne (France), and Naples (Italy). Each country contributed 65 cases on average (range 50–80), for a total of 565 cases. After a quality check, a total of 545 cases were definitively included in the final dataset, that is, 54 cases on average per center (range 44–64).

### 3.2. Dataset Characteristics

**Figure 2:**
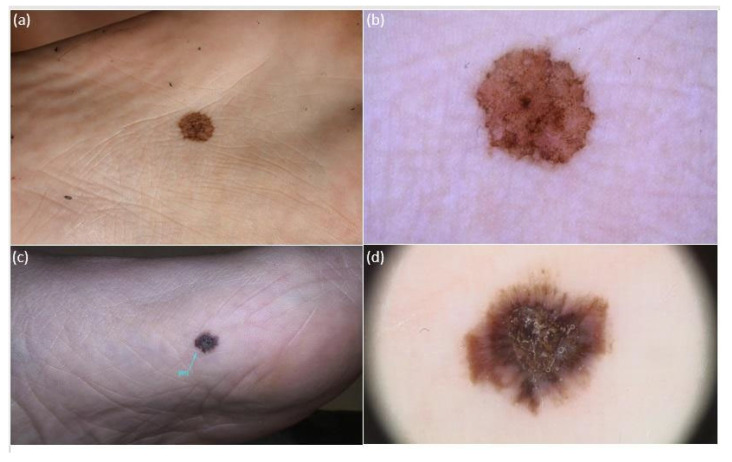
Clinical and dermoscopic (polarized light, 20×) appearance of 2 atypical melanocytic plantar lesions (aMPPLs) of the sole, localized at the central eminence (**a**,**b**) and anterior–medial eminence (**c**,**d**). Both lesions appear as brownish roundish pigmented macules with clear-cut borders and non-homogenous pigmentation, similar diameter and multiple colors, and irregular blotches observed under dermoscopy; however, the lesion of the central eminence was an atypical nevus of 12 mm in a 20-year-old female (**a**,**b**), while the lesion on the anterior–medial eminence was an early melanoma (pt1a) of 13.6 mm in a 63-year-old male (**c**), with additional dermoscopic features of a hyperkeratosic component/blue–white veil and irregular streaks.

**Figure 3:**
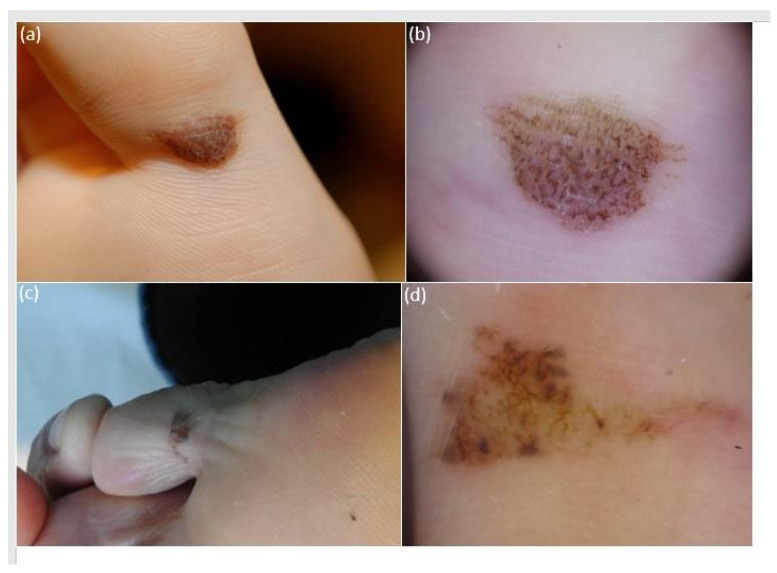
Clinical appearance of atypical melanocytic plantar lesions of the plantar surface of the fingers, namely first (**a**) and fifth (**c**) fingers, presenting as brownish elongated pigmented macules with clear-cut borders and irregular cobblestone-like pigmentation. Lesion one had a maximum diameter of 13 mm and belonged to an 18-year-old female (**a**). Lesion two had a maximum diameter of 11 mm and belonged to a 52-year-old male (**c**). Dermoscopic examination (polarized light, 20×) reveals an overall homogenous color arranged both in a parallel furrow and in a cobblestone (**b**) in case one, which was histologically classified as an acral nevus. Conversely, case two exhibits multiple colors (light brown, dark brown, gray, and reddish) arranged in a multicomponent pattern with streaks, globules, and irregular blotches (**d**); the lesion was histologically classified as an acral melanoma pt1a.

**Figure 4:**
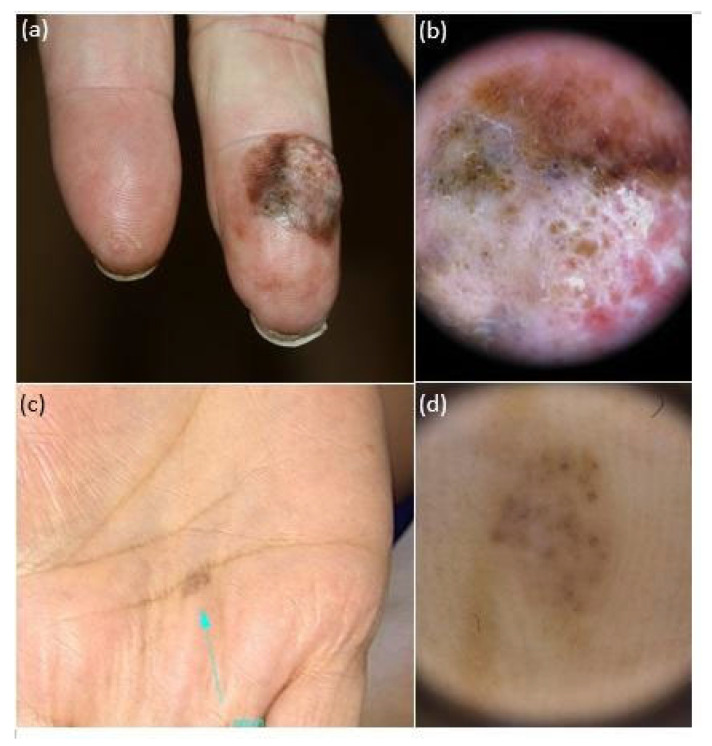
Clinical (**a**,**c**) and dermoscopic (**b**,**d**) appearance of two atypical melanocytic palmar lesions in two women aged 68: the lesion on the volar surface of the finger (**a**) is a multi-colored nodule, with irregular pigmentations and chaotic dermoscopic pattern (**b**), consistent with a histopathologic diagnosis of acral melanoma (pT3a); the lesion on the metacarpal surface appears as a multi-colored macule (**c**, *blue arrow*) with a quite regular dermoscopic pattern and a histopathologic report of acral nevus.

The *iDScore-PalmoPlantar* dataset comprised 542 aMPPL cases with defined histopathological diagnosis and doubtful clinical and dermoscopic appearance, namely 113 (20.8%) melanomas and 429 (79.2%) nevi. Morphologic data of the 542 lesions and patient demographics, anamnestic, and phenotypic data are reported in Table 1, while characteristics of MMs and nevi are reported in Table 2 The analysis of clinical pictures reveals that 291 nevi (67.8%) were flat and 138 (32.1%) were palpable (Figure 2, Figure 3 and Figure 4). 

### 3.3. Lesion Morphological Features

The obtained maximum diameter range for all aMPPLs was 1–50 mm, the average value was 8.83 mm, and the standard deviation was ±7.85 mm (Figure 2, Figure 3 and Figure 4). In melanoma cases, the average diameter was 17.39 (±12.47 standard deviation), range 6–50 mm; in nevi cases, the average diameter was 6.58 (±3.58 standard deviation), range 1–20 mm. The difference between the average diameter of the melanomas and that of the nevi was statistically significant (*p* < 0.001) (Table 2 and Figure 2, Figure 3 and Figure 4).

### 3.4. Lesion Location

#### 3.4.1. All aMPPLs 

Among 542 aMPPL cases, 490 (90.6%) were located on the sole; of them, 98 (87.5%) were MMs and 392 (91.4%) were nevi. A total of 51 out of 542 aMPPL cases (9.4%) were located on the palm, including 14 (12.5%) melanomas and 37 (8.6%) nevi. According to the classification into five macro-areas, aMPPL cases of the sole were predominantly localized on the plantar arch with 229 (46.7%) cases, then on the toes with 111 (22.7%) cases, on the eminence of the sole with 87 (17.8%) cases, and on the heel with 63 (12.9%) cases. On the palm, aMPPL cases were more homogeneously distributed, namely 22 (43.1%) on the palmar medial area, 17 (33.3%) on the finger area, and 12 (23.5%) on the palmar lateral area (Table 1).

#### 3.4.2. Malignant aMPPLs 

On soles, melanomas were prevalent on the plantar arch (29.6%) and heel (27.6%), while on palms, skin distribution was homogeneous among nine subareas (Table 3). Regrouping determined similar proportions of melanomas among four plantar macro-areas and a predominance of malignant cases on the finger area of the palms (Table 2). 

#### 3.4.3. Benign aMPPLs 

On soles, half of the cases were on the plantar arch site (51%), with no significant differences in size to the other seven sites and an unmodified trend after regrouping (Table 2 and Table 4). On palms, a slight predominance was found on the hypothenar surface (27% of cases) (Table 3), but the palmar lateral area (41% of cases) was the most involved after grouping (Table 2).

### 3.5. Patient Data 

#### 3.5.1. Age

Patients with aMPPLs had an age range of 18–92 years. Patients with acral melanoma had an age range from 39 to 92 years old. The difference between the average age of patients with melanomas (65.30 on average (±14.79 sd) and patients with nevi (46.33 on average ± 19.07 sd)) was statistically significant (*p* < 0.001) (Table 1 and Table 2).

#### 3.5.2. Sex

The majority (64%) of patients with aMPPLs were women (347 cases), while men accounted for 36% of cases (i.e., 195). Specifically, this female predominance was sustained by a relevant number (67.4%) of women exhibiting acral nevi (i.e., 289) compared with men (140, 32.6%). Differently, the distribution of acral melanomas was very similar (only a 2% difference) between the two sexes: 51.3% of cases in women, and 48.6% of cases in men. In addition, the difference between the rate of female patients with melanoma and that of female patients with nevi is statistically significant (*p* = 0.002); in males, the two subgroups did not differ significantly (55 melanomas versus 140 nevi) (Table 1 and Table 2). 

### 3.6. Patient Optional Data

A total of 148 cases out of 542 (27.3%) had optional risk factor data assessed (Table 1 and Table 2). The results of distribution analysis according to the histologic diagnosis are reported in Table 2 for those cases in which the optional data regarding patients’ anamnestic/phenotypic and risk factor data were available. 

#### 3.6.1. Anamnestic Data/Risk Factors

Among the available records, the majority of patients with aMPPLs had a negative personal or familial history of melanoma (i.e., melanoma affecting a first-degree relative), which is 14.5% negative versus 2% positive. History of sunburns (>3) in childhood below the age of 14 years was present only in 7.5% of patients, negative in 13.8%, and not assessed in 78.5% of cases. Chronic traumatism was overall not reported on the palms and rarely on the soles (10 patients). In patients with melanomas, no specific anamnestic risk factors reach statistical significance. In patients with nevi, a positive history of sunburns in childhood was reported in 7.9% of cases, and chronic traumatism of soles in 23.3% of cases (Table 1 and Table 2).

#### 3.6.2. Phenotypic Traits

Concerning all patients with aMPPLs, a small proportion (24 cases, 4.4%) had more than 100 common nevi on the body (or more than 10 dysplastic nevi), but the number of missed assessments was relevant (83% of cases) (Table 1). Of them, 21 patients fell in the nevi group (Table 2). 

Phototype III was the prevalent one in this case study (45.7% of patients) (Table 1), as well as in melanoma (68%) and nevi (70%) subgroups (Table 2). The second most prevalent prototype was type II. 

A small number of patients had pheomelanin phenotype, either in the whole case study (6.4%) or in subgroups (7.9% of melanoma patients, 6% of nevi patients).

Only 51 patients were reported to have green/light-blue/blue eyes, with a high rate of non-reporting (76%); of them, 18 had a melanoma and 33 a nevus.

Lastly, a total of 93 patients were reported to have blond hair: 26 with melanoma, and 67 with a nevus. 

### 3.7. Device for Image Acquisition

Table 4 reports in detail how the clinical and dermoscopic pictures for each aMPPL case were obtained. In 31 cases it was not specified what device was used for imaging acquisition (11 melanomas and 20 nevi). Taking into account the whole case study of 542 aMPPL cases, the more frequently employed devices were camera-based systems (46.8% of cases), followed by videodermatoscope (29.5% of cases) and smartphone-based systems (17.8%). 

This trend was similar for the imaging of nevi, with 52% of cases imaged with a camera-based system, 25.4% with a dermatoscope, and 18% with a smartphone. On the contrary, the majority of melanoma cases were imaged with a videodermatoscope (45% of cases) versus 27% with a camera-based system and 17.6% with a smartphone-based system. 

## 4. Discussion

The current knowledge on clinical and epidemiologic features of PP nevi delineates the profile of a small (usually under 6 mm) macule, symmetric in shape and with homogenous pigmentation [9,13,16,38], and mainly derive from Asiatic [4,39,40] or South American countries [4,41,42], with fewer reports from South European countries [2,7,9]. However, equivocal aMPPLs have been poorly or not investigated, especially in Europe [26]. In parallel, large series of acral melanoma in early stages from European populations are lacking, [3,26] due to both low incidence and delayed diagnosis [22,23]. Compared with body or head and neck melanomas, indeed, the diagnosis of PP melanoma is frequently late, with a reported misdiagnosis rate of 20% [3,11,19,20,21,22,23]. A series of factors can be hypothesized to explain this trend: (i) physicians’ reticence to perform biopsies/excision on the sole, which often causes discomfort and painful scar, in addition to nail dystrophy in case of biopsy on the nail apparatus [1,11,19,20,21,22,23]; (ii) immunohistochemical studies and molecular testing that may help to differentiate malignant from benign aMPPLs [1,13,14,43,44] are available only in specific centers, are time-consuming, and require a surgical excision as well; (iii) reflectance confocal microscopy, which is helpful in the non-invasive diagnosis of dermoscopically doubtful cases of the body and face, is not effective on acral skin due to the low penetration [45]; and (iv) patients are sometimes unaware of the onset date of their lesions on the soles (even if they are long-lasting benign nevi). In those cases, the dermatologist should make decisions without the clinical history data, relying on morphological features only; this has a relevant impact on the dermatologist management decision as well, ending up in surgical excision in most cases [22,23,43,44]. From an epidemiological point of view, nevi with mild/moderate/severe atypia and early melanomas on palms and soles are very rare. For this reason, a dataset that collects only PP melanomas at early stages and PP atypical nevi has not previously been set up, to the best of our knowledge. Thus, a better understanding of the aMPPL spectrum is deserved to improve clinical and dermoscopic diagnosis and management [13,23,43,44]. For this purpose, the setting up of a large multicentric European registry dedicated to aMPPLs was needed. Moreover, no specific classification according to plantar and volar subareas has been carried out to date [3,7,9,10,11]. 

The *iDScore-PalmoPlantar* dataset—comprising 542 aMPPL cases coming from 10 different European Centers—is innovative according to several aspects. First, it was designed to provide representative scenarios of the general characteristics of difficult PP lesions that dermatologists have to manage in secondary referring centers. It was indeed balanced to have 20% melanoma and 80% nevi cases and this proportion was chosen in order to reach a compromise between an adequate representation of malignant cases and a reproduction of the epidemiologic in secondary referring centers. Second, the minimum age for inclusion was set at 18 years in order to both exclude pediatric cases a priori (and, consequently, the bias of having large congenital acral nevi in the dataset) and not miss benign lesions in young adults exhibiting chronic traumatism-related alterations. Third, a specifically created classification into 17 subareas was adopted according to anatomical and morphologic criteria (Figure 1) in order to obtain details otherwise missed in previous acral lesion databases. Then, some of the 17 subareas (Table 3) were further grouped into macro-areas following the anatomo-functional criteria of weight bearing, obtaining three macro-areas on the palm and four macro-areas on the sole (Table 1 and Table 4). Fourth, all possible risk factors known or hypothesized for PP melanoma were investigated at contribution time.

Concerning lesion objective data, we found that the maximum diameter value was a significant discriminant (Table 4, *p* < 0.001) between benign and malignant aMPPLs. These data are of main importance as the photographs of benign lesions are homogenously small (6.58 mm on average ± sd3.58), with a number of very equivocal nevi measuring around 12 mm (Figure 2) and few congenital lesions measuring >12 mm (Figure 3), while malignant cases show a larger variation in diameter according to presentation time (17.39 on average ± sd12.47).

The study of lesion location revealed that the hot sites for melanoma of the soles are the plantar arch (29.6%) and heel (27.6%), while those of palmar melanoma are the finger surfaces (50%). 

Benign aMPPLs, instead, were slightly prevalent at hypothenar–metacarpal surfaces of palms, and clearly prevalent on the plantar arch of soles. These data show that there is essentially no difference between weight-bearing/non-weight-bearing areas of the soles in terms of melanoma development, which is in line with recent literature data that excluded a causative role of walking barefoot [16,17,18,46,47,48]. As per palmar melanoma, reports of a trauma occurrence are too very few to derive conclusions. [49] 

According to patient data analysis, age turned out to be a significant discriminant factor for malignancy (Table 4, *p* < 0.001), with patients with PP melanoma generally being older (65.3 years on average) than those with benign aMPPLs (41.33 yrs on average). These data are globally in line with those reported in acral melanoma patients diagnosed in Asia [12], France [3], United States [26], Spain [46], Italy [9], and Korea [47], aged between 59 and 65 years, and those reported from acral nevi patients diagnosed in the United States [4], Italy [26], and Greece [25].

We did not detect any difference in sex distribution for PP melanoma cases (51%F/49%M); this trend is, however, in line with some retrospective studies on acral melanoma patients, reporting distribution of 54%/46%M [46] and 49%/51% [48], while previous monocentric studies showed a slight female or male prevalence (e.g., M:F = 1:1.86 [3], M:F = 1:1.6, [25 M:F = 1:1.9,12 F:M = 1:1.08,26 and M:F = 1:1.712). Precisely, a similar sex distribution was found in patients with cases of early melanoma of the body, in the context of retrospective studies on atypical melanocytic lesions [30,49]. Interestingly, the nevi group of this case study comprised 289 female patients (67,4%F/32.6%M) (Table 4) and the same distribution has been reported in Greek (70%F/30%M) [7], Hispanic (69.5%F/30.5%M) [42], and Italian (63%F/37%M) [9] cohorts of patients screened in secondary referral centers. This repetitive trend may be explained by the fact that women are generally more assiduous in attending skin cancer visits than men. Notably, we previously documented this tendency in multicentric investigations on atypical pigmented lesions of the face [31,34] and trunk [30,49]. 

The descriptive and association analysis of patient anamnestic data, risk factors, and phenotypic traits showed that the aMPPL population was essentially homogeneous, with no significant difference between benign and malignant cases. This can be first explained by the high rate of non-assessment for the majority of patients, mostly ascribable to the retrospective collection performed by participating centers; then, by the difficulty in defining the entity of the traumatism or sunburn by patients themselves; and lastly, by the fact that recent meta-analysis failed to confirm the hypothesis on a clear causative role of injuries/trauma or history of sunburns in infancy in acral melanoma development [3,42,44,46,50,51,52]. Indeed, the current evidence from laboratory studies suggests that PP acral melanoma development seems to arise in a certain cancer susceptibility setting (which is, however, different from the renowned genetic signature of melanoma families) and does not follow the classic risk factors addressed for body and face melanomas [3,13,14,42,44,46,47].

At last, the results of the imaging device analysis showed that there was a slight tendency to use the camera-based devices to photograph benign aMPPL lesions, while the majority of malignant aMPL case images were acquired with a videodermatoscope. These frequencies of use essentially reflect the equipment of each center, but a series of considerations can be raised. It can be argued that camera-based/smartphone-based methods may be preferred to acquire pictures of potentially benign lesions of the soles because they are more handy/rapid to use, whereas fixed videodermatoscope devices may be a bit uncomfortable/time-consuming, especially in front of elderly patients in standing position, and are best reserved for the ugliest lesions, in which a larger screen is required [53,54,55,56]. 

Limitations of this study to take into account are the following: (i) since all cases have been histologically analyzed, there is an intrinsic selection bias based on the excisional criteria; (ii) the majority of patients were identified as phototype III, generating a potential bias in phenotypic data analysis; and (iii) the sample size of palmar lesions was small due to the very low incidence of palmar melanoma in Europe.

## 5. Conclusions

The creation of the integrated PP dataset and the analyses carried out (descriptive and univariate) are devoted to a better understanding of aMPPLs in the European population where they are poorly investigated and represent phase I of the *iDScore PalmoPlantar* project. Indeed, by combining both the morphological features and the patient data, we aimed to delineate some recurrent patterns of Caucasian patients with aMPPLs frequently attending skin cancer screening centers. In general, in a patient aged >50 years exhibiting an aMPPL larger than 8 mm on the heel/plantar arch or fingers of the hand, the risk of melanoma is very high, independent of sex. If a patient older than 65.3 years presents with a lesion larger than 17 mm, palpable, immediate excision should be performed with large margins. Then, if a patient aged up to 49 years has a flat lesion of up to 7 mm in diameter localized to the palmar/hypothenar or thenar surface of the palms or the plantar arch of the sole, we can be quite confident that it is a benign aMPL. However, these preliminary data need to be confirmed on a larger dataset, especially for palmar melanoma cases, during the next decades. Moreover, further investigations for the *iDScore-PalmoPlantar* project will be carried out to combine and interpret these data according to the dermoscopic analysis and the detailed localization/distribution analysis. Briefly, phase II of the *iDScore-PalmoPlantar* project will consist of obtaining the average pattern analysis values of all the collected cases based on the consensus of two out of three dermoscopists variously skilled, for a total of 156 tele-dermoscopic investigations across Europe [32,33,34,36]. Finally, in phase III, the large amount of data obtained in the two previous phases will undergo multivariate analysis (i.e., forward–backward stepwise logistic regression) in order to select a pool of interdependent significant parameters useful to the setting up of a scoring system Bayesian classifier. This risk checklist, named the *iDScore-PalmoPlantar* model, will be able to provide an aMPPL score between 0 (no risk) and 15 (100% risk of malignancy). The management suggestions will be derived from the risk ranges estimate (i.e., mild, moderate, high, or very high), with the score threshold estimated by the leave-one-out technique using the variation in the area under the ROC curve [27,28,29,30,31,32]. The ultimate goal of the present dataset is the development of an integrated clinic–anamnestic–dermoscopic *iDScore-PalmoPlantar* model to help clinicians—in real time—in orienting their diagnostic suspects in front of difficult atypical PP lesions and to support them in management decisions of no/long/short follow-up or excision. In the next future, also a DCNN (deep convolutional neural network) [57] based model could be derived from the *iDScore-PalmoPlantar dataset* [58,59]. 

## Figures and Tables

**Table 1:** Characteristics of the case study of 542 atypical melanocytic palmoplantar lesions (aMPPLs) comprising the *iDScore-PalmoPlantar* dataset.

Lesion Data	n (%)/Mean ± SD		
**Histological Diagnosis**	542		
*Nevus*	429 (79.2%)		
*Malignant melanoma*	113 (20.8%)		
Maximum diameter	8.83 ± 7.85		
Four macro-areas of the sole	490 (90.6%)		
• Toe area *(fingers + interdigital spaces + lateral surface of the fingers)*	111 (22.7%)		
• Eminence of the sole area *(anterior lateral + central + anterior medial eminence of the sole)*	87 (17.8%)		
• *Plantar arch area*	229 (46.7%)		
• *Heel area*	63 (12.9%)		
Three macro-areas of the palm	51 (9.4%)		
• Palmar lateral area *(proximal phalangeal surface + thenar eminence)*	12 (23.5%)		
• Fingers *(lateral surface of the fingers + fingertips + interdigital spaces)*	17 (33.3%)		
• Palmar medial area *(hypothenar surface + metacarpal surface + central metacarpal)*	22 (43.1%)		
**Patient Data**			
Age	46.33 ± 19.07		
Male	195 (36.0%)		
Female	347 (64.0%)		
ANAMNESTIC DATA/RISK FACTORS	YES	NO	NA
Personal/family history of melanoma—1st-degree-relative	11 (2.0%)	79 (14.5%)	452 (83.4%)
History of sunburns (>3) in childhood below the age of 14 years	41 (7.5%)	75 (13.8%)	426 (78.5%)
Chronic traumatism of palms	0 (0.0%)	7 (12.9%)	44 (86.3%)
Chronic traumatism of soles	10 (1.8%)	138 (25.4%)	342 (63.0%)
PHENOTYPIC TRAITS			
Presence of >100 common nevi or >10 dysplastic nevi on the body	24 (4.4%)	67 (12.3%)	451 (83.2%)
Phototype	355 (65.5%)		187 (34.5%)
• II	94 (17.3%)		
• III	248 (45.7%)		
• IV	11 (2.0%)		
• V	2 (0.3%)		
Pheomelanin phenotype	35 (6.4%)	65 (11.9%)	442 (81%)
Presence of green/light blue/blue eyes	51 (9.4%)	76 (14%)	415 (76%)
Presence of blond hair	93 (17.1%)	69 (12.7%)	380 (70.1%)

**Table 2:** Distribution of anamnestic and phenotypic data of 542 atypical melanocytic palmoplantar lesion (aMPPL) cases, grouped according to seven macro-areas (four on the sole and three on the palm).

	n (%)/Mean ± SD		
Lesion Data	MMs (113)	Nevi (429)	*p*
Maximum diameter	17.39 ± 12.47	6.58 ± 3.58	<0.001
Body site			0.285
*Four macro-areas of the sole* *	98 (87.5%)	392 (91.4%)	
• *Toe area*	21 (21.4%)	90 (23.0%)	
• *Eminence of the sole area*	21 (21.4%)	66 (16.8%)	
• *Plantar arch area*	29 (29.6%)	200 (51.0%)	
• *Heel area*	27 (27.6%)	36 (9.2%)	
*Three macro-areas of the palm* ^#^	14 (12.5%)	37 (8.6%)	
• *Palmar medial area*	3 (21.4%)	9 (24.3%)	
• *Finger area*	7 (50.0%)	10 (27.0%)	
• *Palmar lateral area*	4 (28.6%)	18 (41.6%)	
**Patient Data**			
Age	65.30 ± 14.79	41.33 ± 16.81	<0.001
Male	55 (48.6%)	140 (32.6%)	
Female	58 (51.3%)	289 (67.4%)	0.002
Anamnestic Data/Risk Factors			
Personal/family history of melanoma—1st-degree relative			0.520
No	9 (7.9%)	70 (16.3%)	
Yes	0 (0.0%)	11 (2.5%)	
History of sunburns (>3) in childhood below the age of 14 years			
No	25 (22.1%)	50 (11.6%)	
Yes	7 (0.6%)	34 (7.9%)	
Chronic traumatism on soles			
No	15 (13.2%)	129 (30.0%)	
Yes	0 (0%)	10 (23.3%)	
Chronic traumatism on palms			
No	10 (8.8%)	94 (21.9%)	
Yes	0 (%)	1 (0.2%)	
PHENOTYPIC TRAITS			
Presence of >100 common nevi or >10 dysplastic nevi			1.000
No	7 (6.1%)	60 (13.9%)	
Yes	3 (2.6%)	21 (4,8%)	
Phototype (%)	100%	100%	0.717
II	19 (29.7%)	75 (25.8%)	
III	44 (68.8%)	204 (70.1%)	
IV	1 (1.6%)	10 (3.4%)	
V	0 (0.0%)	2 (0.7%)	
Pheomelanin phenotype			
No	17 (15.0%)	48 (11.1%)	0.610
Yes	9 (7.9%)	26 (6.0%)	
Presence of green/light blue/blue eyes			
No	23 (20.3%)	53 (12.3%)	0.320
Yes	18 (15.9%)	33 (7.6%)	
Presence of blond hair			
No	14 (12.3%)	55 (12.8%)	0.430
Yes	26 (23%)	67 (15.6%)	

* SOLE SUBAREAS: Toe area (plantar surface of the fingers + lateral surface of the fingers + interdigital spaces); eminence of the sole area (anterior eminence + central eminence + antero-medial eminence); plantar arch area; and heel. ^#^ PALM SUBAREAS: Finger area (fingertips + lateral surface of the fingers + interdigital spaces + volar surface of the finger + proximal phalangeal surface); palmar lateral area (metacarpal area + hypotenar); and palmar medial (thenar + central metacarpal).

**Table 3:** Distribution of 542 atypical melanocytic palmoplantar lesions (aMPPLs) of the *iDScore-PalmoPlantar* dataset according to histologic diagnosis and detailed body location to 8 plantar and 8 palmar subareas.

Lesion Data	aMPPLs n = 542	MMs n = 113	Nevi n = 429
***Eight Subareas of the sole***	490	98 (87.5)	392 (91.4)
Anterior lateral eminence of the sole	21 (3.18%)	3 (3.1%)	18 (4.6%)
Anterior medial eminence of the sole	45 (7.85%)	15 (15.3%)	30 (7.7%)
Central eminence of the sole	21 (4.46%)	3 (3.1%)	18 (4.6%)
Heel	63 (1.21%)	27 (27.6%)	36 (9.2%)
Interdigital spaces (foot)	34 (5.73%)	4 (4.1%)	30 (7.7%)
Lateral surface of the fingers (foot)	36 (7.21%)	8 (8.2%)	28 (7.1%)
Plantar arch	229 (43.9%)	29 (29.6%)	200 (51.0%)
Plantar surface of the fingers	41 (7.85%)	9 (9.2%)	32 (8.2%)
***Nine Subareas of the palms***	36	14 (12.5%)	37 (8.6%)
Central metacarpal	2 (5.6%)	0 (0.0%)	2 (5.4%)
Fingertips (hand)	2 (5.6%)	2 (14.3%)	0 (0.0%)
Interdigital spaces	3 (8.3%)	0 (0.0%)	3 (8.1%)
Ipothenar surface	11 (30.6%)	1 (7.1%)	10 (27.0%)
Lateral surface of the fingers (hand)	5 (13.9%)	3 (21.4%)	2 (5.4%)
Metacarpal surface	11 (30.6%)	3 (21.4%)	8 (21.6%)
Thenar surface	10 (27.8%)	3 (21.4%)	7 (18.9%)
Volar surface of the fingers	7 (19.4%)	2 (14.3%)	5 (13.5%)
Proximal phalangeal surface	0	0	0

**Table 4:** Characterization of digital imaging acquisition of 542 atypical melanocytic palmoplantar lesion (aMPPL) cases of the *iDScore-PalmoPlantar* dataset: three different devices for dermoscopic imaging acquisition are reported, along with the distribution per histologic diagnosis.

Device Type Used for Image Acquisition/	aMPPLs	Melanomas	Nevi
**n (%)**	542 (100%)	113 (100%)	429 (100%)
Camera-based system	254 (46.8%)	31 (27.4%)	223 (51.9%)
Videodermatoscope	160 (29.5%)	51 (45.1%)	109 (25.4%)
Smartphone-based system	97 (17.8%)	20 (17.6%)	77 (17.9%)
Unknown/unspecified	31 (5.7%)	11 (9.7%)	20 (4.6%)

## Data Availability

Data are available from the corresponding author upon reasonable request.
